# Sodium Butyrate Induces Growth Inhibition and Apoptosis in Human Prostate Cancer DU145 Cells by Up-Regulation of the Expression of Annexin A1

**DOI:** 10.1371/journal.pone.0074922

**Published:** 2013-09-23

**Authors:** Dawei Mu, Zhuo Gao, Heqing Guo, Gaobiao Zhou, Bin Sun

**Affiliations:** 1 Department of Urology, Air Force General Hospital, Beijing, China; 2 Department of Nephrology, Air Force General Hospital, Beijing, China; Roswell Park Cancer Institute, United States of America

## Abstract

**Background:**

Sodium butyrate, a histone deacetylase inhibitor, has emerged as a promising anticancer drug for multiple cancers. Recent studies have indicated that sodium butyrate could inhibit the progression of prostate cancer; however, the exact mechanism is still unclear. The aim of this study was to investigate the mechanism of sodium butyrate action in prostate cancer DU145 cells.

**Methods:**

The inhibitory effects of NaB on cell growth were detected by the 3-(4, 5-dimethylthiazol-2-yl)-2, 5-diphenyltetrrazolium bromide assay. Cell apoptosis was determined by flow cytometric analysis of DU145 cells stained with annexin V and PI. Hoechst 33258 and fluorescence microscopes were used to observe the nuclear morphology of DU145 cells after treatment with NaB. ANXA1 knockdown cells were established through transfection with ANXA1 siRNA. ANXA1 mRNA levels were measured by qRT-PCR. Bcl-2, Bax, ANXA1, ERK1/2 and pERK1/2 were detected by western blot.

**Results:**

NaB significantly inhibited the growth and induction apoptosis of DU145 and PC3 cells in a dose-dependent manner. Expression of the anti-apoptosis gene Bcl-xl and Bcl-2 in DU145 cells are decreased and expression of the pro-apoptosis gene Bax and Bak increased after NaB treatment. Further studies have demonstrated that NaB up-regulated the expression of ANXA1 and that the tumor inhibition action of NaB was reduced markedly through knockdown of the ANXA1 gene in DU145 cells. Moreover, the siANXA1 cells showed that cell proliferation increased and cell apoptosis was induced by the inactivation of extracellular regulated kinase (ERK).

**Conclusion:**

Our results support a significant correlation between NaB functions and ANXA1 expression in prostate cancer, and pave the way for further studying the molecular mechanism of NaB actions in cancers.

## Introduction

Prostate cancer is a relatively common malignant cancer and the second most commonly diagnosed cancer in men [1]. Recently, several effective therapies can be offered for the clinical treatment of prostate cancer; such as surgery (radical prostatectomy) and radiotherapy (external-beam radiotherapy, brachytherapy, or both) for patients with early-stage disease and adjuvant systemic treatments (chemotherapy), which are effective for advanced-stage disease [2]; However, the therapies all have different side-effect profiles. Sodium butyrate, one of the most widely studied histone deacetylase inhibitors, has emerged as a promising anticancer drug by altering gene expression through chromatin modification [3].

Sodium butyrate, the sodium salt of butyric acid, has been reported to have a wide variety of effects on cultured mammalian cells, such as the induction of differentiation and inhibition of cell proliferation, at relatively low concentrations [4], [5]. Moreover, there is a body of evidence that shows that sodium butyrate could induce apoptosis in numerous cancer cells [6], [7]. Due to its growth-inhibiting and apoptosis-inducing activity, sodium butyrate, alone or in combination with other anti-cancer drugs, could be used to treat a number of malignant tumors [8], [9]. Recently, Degui Wang and colleagues demonstrated that sodium butyrate can inhibit the proliferation of human prostate cancer cell lines and has a synergetic effect with anticancer drugs in treating prostate cancer both *in vitro* and *in vivo* [10]. The clinical utility of sodium butyrate is restricted by its mechanism of action, which is still unclear. An important recent advance has been that sodium butyrate has been found to up-regulate the expression of annexin A1 (ANXA1) in human colon adenocarcinoma cells [11].

Annexins are a family of phospholipid and calcium binding proteins which are comprised of 12 members in mammals. Annexins are omnipresent and expressed in a variety of organisms [12]. There is some evidence that annexin family members have important roles in tumoral development and progression because these proteins can affect the invasiveness and proliferation of cancer cells and show dysregulation in numerous cancers [13]. ANXA1, the first member of the family of annexins, is an intracellular protein which plays important roles in apoptosis, proliferation, and cancer [14], [15]. A great deal of controversy still exists regarding the expression of ANXA1 in different types of cancers. Several studies have shown that the expression of ANXA1 is down-regulated in cervical, breast, head and neck, or thyroid cancer [16], [17], [18], [19], [20]. On the other hand, there is also some evidence that an increase of ANXA1 expression has occurred in other types of cancer, such as pancreatic, esophageal, and gastric carcinomas [21], [22], [23]. There is a lack of adequate functional experimental evidence clearly defining the role of annexins in prostate cancer. Several recent studies have indicated that annexin II was reduced in prostate cancers and annexins A1 and A2 have already been associated with tumor suppression in prostate cancer [24]. Lecona and colleagues demonstrated that butyrate could up-regulate the expression of ANXA1 in human colon adenocarcinoma cells [11]. These studies highlight the possible role of sodium butyrate as an ANXA1 regulator to inhibit the progression of prostate cancer. The study provides the first direct evidence in prostate cancer cells that sodium butyrate can up-regulate the expression of ANXA1 leading to cell apoptosis. We show that sodium butyrate inhibits cell growth and induces cell apoptosis in prostate cancer cells by up-regulating the expression of ANXA1 through the ERK signaling pathway.

## Materials and Methods

### Antibodies

Mouse anti-Bcl-2 monoclonal antibody, mouse anti-Bax monoclonal antibody, mouse anti-Bcl-xl monoclonal antibody, mouse anti-Bak monoclonal antibody, mouse anti-ANXA1 monoclonal antibody and mouse anti-β-actin monoclonal antibody were obtained from Abcam (Cambridge, MA). Anti-phospho-ERK (pERK) monoclonal antibody, anti-ERK polyclonal antibody and HRP-conjugated goat anti-mouse IgG were obtained from Santa Cruz Biotechnology (Santa Cruz, CA).

### Cell Culture

The human prostate cancer cellular line DU145 cells (HTB-4; ATCC, Rockville, MD) and PC3 cells (ATCC, Rockville, MD) were cultured at 37°C under a humidified atmosphere of 5% CO_2_ and 95% air and were grown in RPMI 1640 medium (Gibco BRL, Gaithersburg, MD) supplemented with 10% fetal bovine serum (Gibco BRL, Gaithersburg, MD), 1% penicillin-streptomycin and 1% glutamine.

### Cell Viability Assay

The cell viability was determined by the Trypan Blue exclusion assay, as described previously with minor modifications [25]. Briefly, DU145 and PC3 cells were plated separately in 24-well plates at 2×10^5^ cells per well, grown in RPMI 1640 medium for 24 h and then added to different concentrations of NaB to a final concentration of 0, 1, 5, and 10 mmol. Only dimethyl sulfoxide (DMSO) was added for the control group. Cells were grown in an incubator for different periods. Cell suspensions (10 µL) in PBS were mixed with 40 µL Trypan Blue (Sigma, St. Louis, MO). The cells were subsequently washed three times with DMSO before counting under a light microscope. Cells demonstrating dye uptake were classed as nonviable.

### MTT Proliferation Assay

DU145 and PC3 cell proliferation were measured using the previously described 3-(4,5-dimethylthiazol-2-yl)-2,5-diphenyltetrrazolium bromide (MTT) assay [26]. Briefly, DU145 and PC3 cells (approximately 2×10^5^) were plated separately in 24-well plates for 48 h. The MTT (20 µL, 5 mg/mL) were then added into the DU145 and PC3 cancer cells for 4 h at 37°C. DMSO (200 µL) was added to solubilize the crystals for 25 min at room temperature. The optical density (OD) value was measured at a wavelength of 490 nm by a spectrophotometer (Multiskan MK3, Thermo, Waltham, MA). All experiments were performed at least three times and were calculated using average results.

### Detection of Apoptotic Cells by Flow Cytometry

The cell apoptotic ratio was measured by annexin V-FITC and PI staining followed by analysis with flow cytometry (Beckman-Coulter, Brea, CA). Briefly, 2×10^5^ cells per well were plated in 24-well plates and then various concentrations of NaB were added. Cells were trypsinized and harvested by centrifugation and then incubated with Annexin V and PI for 15 min at room temperature. Apoptosis was examined by flow cytometry using Annexin V-FITC/PI kit (BD PharMingen, San Diego, CA). After 30 minutes at 37°C the cells were ready for the analysis by the flow cytometry and the cell apoptotic ratio was determined.

### Nuclear Morphological Observation of NaB Treated DU145 Cancer Cells

Cells were plated in 24-well plates at 2×10^4^ cells per well and treated with NaB at various concentrations. Cells were fixed using 10% buffered formalin/4% formaldehyde, and were then washed twice with PBS and stained with Hoechst 33258 staining solution. The nuclear morphology of DU145 cells was observed by reflected fluorescence microscope (Nikon MF30 LED, Japan). Quantification of apoptotic cells was performed by counting 500 DU145 cells, and then determining the percentage of apoptotic cells in at least five cross-sections per slide. The experiment was repeated at least three times.

### Quantitative Real-time -PCR (qRT-PCR) Assay

*ANXA1* mRNA levels were measured in prostate cancer cell lines DU145 using the qRT-PCR method. Total cellular RNA was isolated from DU145 cells using Trizol Reagent (Invitrogen, Carlsbad, CA) according to the manufacturer’s protocol. 2 µg of total RNA was subjected to reverse transcription using a High-Capacity RNA-to-cDNA Kit (Applied Biosystems) according to the manufacturer’s instructions. QRT-PCR was performed in a BioRad MyIQ single-color real-time PCR detection system using SYBR Green Supermix (BioRad, Carlsbad, CA). The primer sequences used for qRT-PCR were as follows: *ANXA1* forward, GAGCCCCTATCCTACCT TCA; *ANXA1* reverse, GGTTGCTTCATCCACACCT; *ACTIN* forward, TCCCTGGAGAAGAGCTACGA; *ACTIN* reverse, AGGAAGGAAGGCTGGAAGAG. These primers were all synthesized by Sangon Biotech Co., Ltd. (Shanghai, China). Cycling conditions were as follows: pre-incubation: 95°C, 10 min; PCR: 94°C, 10 s and 60°C, 30 s, 45 cycles; final elongation: 72°C, 10 min. Expression levels of the relative genes were calculated using the 2^−ΔΔC^T method [27] and with the β-actin mRNA as an internal control.

### Western Blot Assay

The total protein of DU145 cells were extracted by using RIPA lysis buffer (Beyotime, Nantong, China) according to the operating protocols. The protein concentration in the lysates was determined by BCA protein assay kit (Beyotime, Nantong, China). The proteins (40 µg/lane) were subjected to 10% SDS-PAGE and electrophoretically transferred to Immobilon P Millipore (Bedford, MA, USA). Mouse monoclonal and polyclonal antibodies were used as the primary antibody and HRP-conjugated goat anti-mouse IgG was used as the secondary antibody. The bound antibodies were visualized using LumiGLo reagent (Pierce, Rockford, IL) and the levels of the proteins relative to β-actin were determined. All experiments were repeated at least three times.

### Small Interfering RNA Transfection

The prostate cancer DU145 cells with the ANXA1 protein were transfected with ANXA1 siRNA (siANXA10) or the control siRNA (siMock) (Takara, Dalian, China) using Lipofectamine 2000 (Invitrogen, Carlsbad, CA) according to the method of Maxime [28], with minor modifications. Briefly, a day before transfection, 2×10^5^ prostate cancer cells were seeded on a 60 mm plates in RPMI 1640 containing 5% FCS. In total, 1.5 µg of plasmid DNA was transfected into DU145 cells for RNA and protein extraction. Stable transfectants of DU145 cells were selected with the culture medium containing puromycin (Invitrogen, Carlsbad, CA). The viable colonies were picked up and transferred to new dishes after 2 to 3 weeks after transfection. The siANXA1 target sequences were: 5′-ATGCCTCACAG- CTATCGTGAA-3′ (sense), and 5′-TTCACGATAGCTGTGAGGCAT-3′ (anti-sense). SiANXA1-transfected and siMock-transfected cells were used for further experiments.

### Statistical Analysis

All data were expressed as mean ± the standard error of the mean (SEM). The differences between control and NaB treated groups were compared by Dunnett’s test subsequent to ANOVA. A value of P<0.05 was considered statistically significant. All experiments were repeated at least three times.

## Results

### NaB Inhibits Proliferation and Cell Survival in DU145 Cells

**Figure 1:**
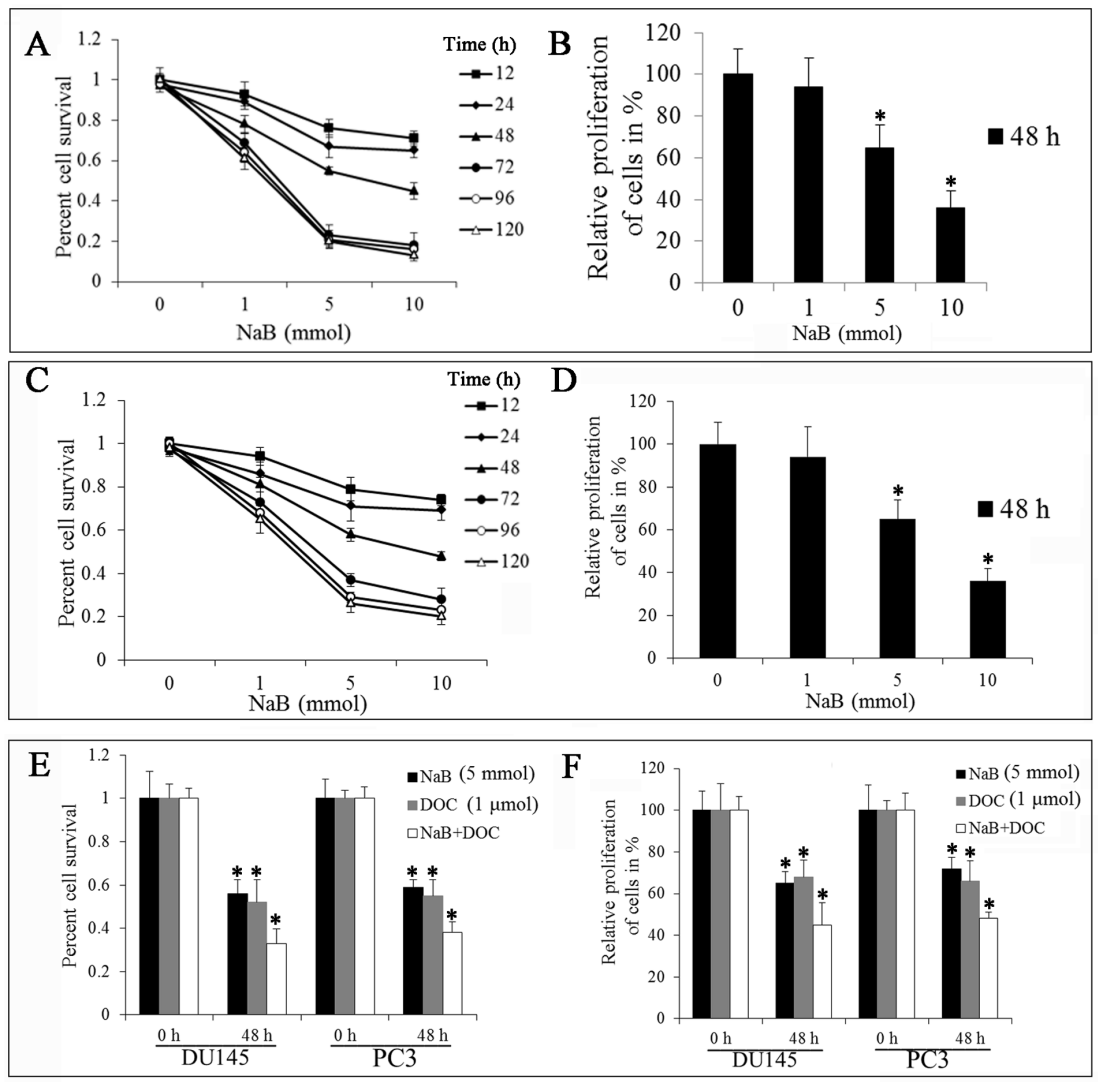
NaB inhibits cell viability and proliferation of human prostate cancer DU145 cells. (A) NaB inhibits cell viability in a time- and dose-dependent manner in DU145 cells. Cancer cells were treated with medium alone or various concentrations of NaB (1, 5, 10 mmol) for various time (12, 24, 48, 72, 96 and 120 h). (B) Effect of NaB on DU145 cell proliferation. Cancer cells were treated with medium alone or various concentrations of NaB (1, 5, 10 mmol) for 48 h. (C) NaB inhibits cell viability in PC3 cells. Cancer cells were treated with medium alone or various concentrations of NaB (1, 5, 10 mmol) for various time (12, 24, 48, 72, 96 and 120 h). (D) Effect of NaB on PC3 cell proliferation. Cancer cells were treated with medium alone or various concentrations of NaB (1, 5, 10 mmol) for 48 h. (E) Effect of NaB in combination with DOC on the cell viability of DU145 and PC3 cells. Cancer cells were treated with medium alone or NaB (5 mmol) in combination with DOC (1 µmol) for 48 h. (F) Effect of NaB in combination with DOC on DU145 and PC3 cell proliferation. Cancer cells were treated with medium alone or NaB (5 mmol) in combination with DOC (1 µmol) for 48 h. Cell viability was determined by Trypan Blue exclusion assay. Cell proliferation was detected by MTT assay. NaB significantly inhibited the growth of DU145 and PC3 cells. All experiments were repeated at least three times. Data are means ± SEM. (n = 3) (Dunnett’s test, *P<0.05 *vs.* control).

The cytotoxic effect of NaB on human prostate cancer DU145 and PC3 cells were examined with varying concentrations of NaB and times by Trypan Blue exclusion assay (Fig. 1 A and C). The results showed that the viability of DU145 and PC3 cells were all reduced by NaB in a dose- and time-dependent manner. NaB induced an apparent decrease in the number of viable host cells dose-dependently with an IC_50_ of 7.07 mmol/L at 48 h in DU145 cells and IC_50_ of 8.71 mmol/L at 48 h in PC3 cells. A dose range of 0–10 mmol NaB treating prostate cancer DU145 cells and PC3 for 48 h are our expected concentration and time.

We also measured the effect of NaB on DU145 and PC3 cell proliferation by MTT assay. As shown in Fig. 1B and D, there was a drastic decrease in the proliferation of cells with increasing doses of NaB (0–10 mmol). NaB inhibited cell proliferation dose-dependently in both DU145 and PC3 cells(Fig. 1B and D). These observations indicate that NaB can inhibit the proliferation and survival of DU145 and PC3 cells, however, DU145 is more sensitive for NaB treatment.

There has some evidence that NaB combined with other anticancer agents may be very useful in bladder cancers [10]. Thus, we examined the effect of NaB in combination with docetaxel (DOC) on prostate cancer cells. The results showed that there is a synergistic inhibition of human prostate cancer cell growth by NaB and DOC (Fig. 1E). To further confirm the synergistic growth inhibition effect of NaB, the cell proliferation was detected by MTT assay. As shown in Fig. 1F, NaB combined with DOC could significant inhibit the cell proliferation.

### NaB Induces Apoptosis in DU145 Cell Lines

**Figure 2:**
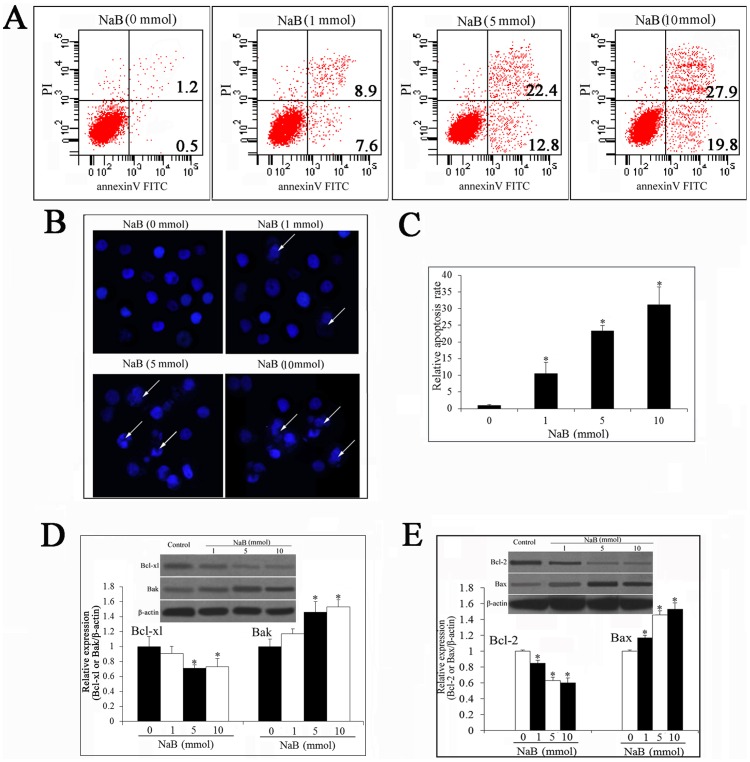
NaB treatment induces apoptosis of DU145 cells in a dose-dependent manner. (A) Flow cytometry analysis of NaB treated DU145 cells stained with annexin V and PI. (B) Nuclear morphological observation of NaB treated DU145 cancer cells. Hoechst 33258 and a fluorescence microscope were used to observe the nuclear morphology of DU145 cells after treatment with NaB (400×). (C) Quantitative analyses of the rate of apoptosis cell. (D) NaB modulates expression of Bcl-xl and Bak in DU145 cells. (E) NaB modulates expression of Bcl-2 and Bax in DU145 cells. The expression of Bcl-xl, Bcl-2, Bax and Bak were determined by western blot analysis. All experiments were repeated at least three times. Data are means ± SEM. (n = 3) (Dunnett’s test, *P<0.05 *vs.* control).

The induction of apoptosis by NaB was first examined by flow cytometric analysis of DU145 cells stained with annexin V and PI (Fig. 2A). The results showed that NaB caused a dose-dependent increase in cell apoptosis, and the apoptosis rate increased to 30% when the concentration of NaB was over 5 mmol/L.

We next quantified the changes in cell nuclear morphology by Hoechst staining under fluorescence microscopy. As shown in Fig. 2B, the cancer cells showed bright fluorescent nuclei blebbing and DNA fragmentation, compared with the control DU145 cells after treatment with NaB for 48 hours. Moreover, the number of apoptotic cells was increased in a NaB dose-dependent manner (Fig. 2C). Simultaneously, the cell density was decreased in a dose-dependent manner. These changes are in accordance with the characteristics of apoptosis.

Cell apoptosis appears to be controlled by numerous genes; *Bcl-xl, Bcl-2*, *Bax and Bak* are the most important apoptosis-related genes among them. Bcl-xl and Bcl-2 protein are two cell death inhibitors, whereas Bax and Bak have a critical role in facilitating apoptosis. Thus, we measured the expression of Bcl-xl, Bcl-2, Bax and Bak proteins in DU145 cells by western blot after treatment with NaB (Fig. 2D and E). The results showed that the expression of Bcl-xl and Bcl-2 were decreased and the expression of Bax and Bak were simultaneously up-regulated. The apoptosis rate was significantly increased because of the up-regulation of pro-apoptosis genes.

### NaB Up-regulates the Expression of ANXA1 in Prostate Cancer DU145 Cell Line

**Figure 3:**
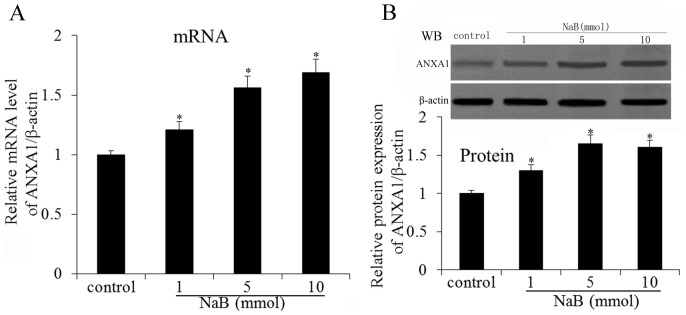
NaB treatment up-regulates the expression of ANXA1 in DU145 cells. (A) ANXA1 mRNA expression is detected by qRT-PCR. NaB treatment groups significantly higher than control group (treatment by medium only). (B) Western blot analysis of ANXA1 expression in DU145 cells after NaB treatment. Data are normalized with β-actin values and are expressed as fold changes of β-actin in NaB treated group relative to control. All experiments were repeated at least three times. Data are means ± SEM. (n = 3) (Dunnett’s test, *P<0.05 *vs.* control).

The above results indicated that NaB could inhibit proliferation and induced apoptosis in prostate cancer cells; however, the mechanism of NaB action is still unclear. Lecona and colleagues suggested that butyrate can up-regulate the expression of annexin A1 in human colon adenocarcinoma cells. Thus, we questioned whether there was some connection between NaB and ANXA1. The expression of ANXA1 and the up-regulation of ANXA1 by NaB in DU145 cells were determined by RT-PCR and western blot, respectively (Fig. 3). As shown in Fig. 3, increased ANXA1 mRNA levels and protein expression were detected in the DU145 cells treated with NaB. The results demonstrated that NaB could up-regulate the expression of ANXA1 in DU145 cells.

### Establishment and the Functional Analyses of ANXA1 Knockdown Cells

**Figure 4:**
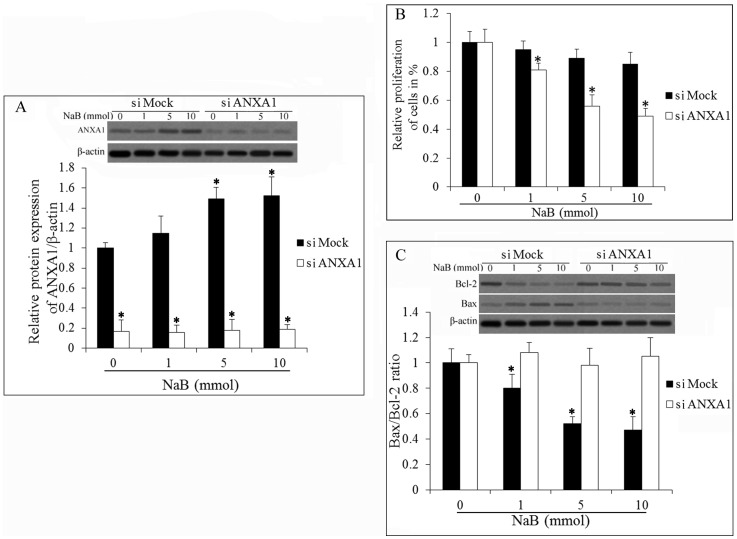
The effect of NaB treatment on siANXA1-transfected cells. (A) Western blot analysis of ANXA1 expression in siANXA1 and siMock cells after NaB treatment. Data were normalized with β-actin values and are expressed as fold changes of β-actin in NaB treated group relative to control. (B) The proliferation of siANXA1 and siMock cells after NaB treatment. (C) Western blot analysis of the expression of Bax and Bcl-2 in siANXA1 and siMock cells. Bcl-2 and Bax expression levels were normalized to β-actin and are presented as the Bax/Bcl-2 ratio. All experiments were repeated at least three times. Data are means ± SEM. (n = 3) (Dunnett’s test, *P<0.05 *vs.* control).

Since ANXA1 expression was up-regulated in the DU145 cells treated with various concentrations of NaB, we assumed that ANXA1 might play a significant role in the activity of NaB. To measure the ANXA1 functions *in vitro,* a siRNA experiment was performed in DU145 cells. The mRNA level and protein ANXA1 in the siANXA1-transfected group were significantly lower than the siMock transfected group (Fig. 4A). We next examined the effect of ANXA1 knockdown on the activity of NaB, including the inhibition of cell growth (MTT assay) and the induction of cell apoptosis (western blot assay). The results showed that there was an increase in cellular growth of the siANXA1-transfected group compared with the siMock-transfected group (Fig. 4B). Moreover, the apoptosis rate was decreased in the siANXA1-transfected cells (Fig. 4C). The results indicated that the NaB activity of growth inhibition and pro-apoptosis in DU145 cancer cells was associated with the over-expression of ANXA1.

### Over-expression of ANXA1 is Directly Related to the Activation of ERK

**Figure 5:**
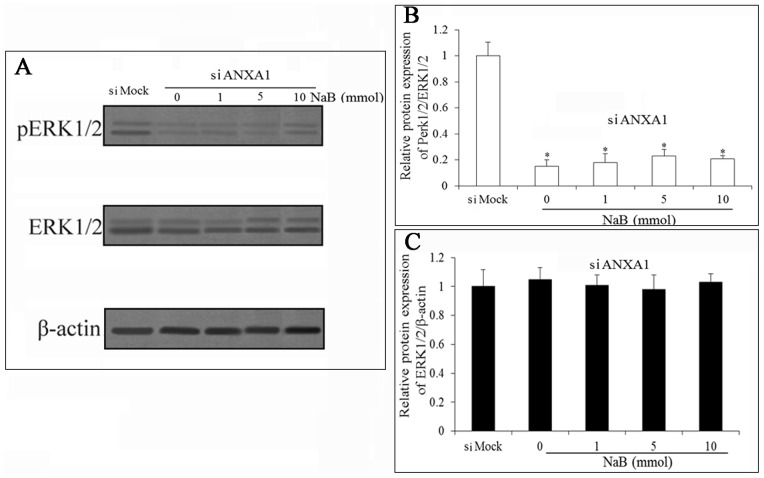
ANXA1 knockdown inhibits ERK activation. The expression of p-ERK and ERK are detected by western blot analysis. The expression of p-ERK is reduced markedly in ANXA1 knockdown cells compared with the siMock cells, whereas the ERK level is almost unchanged. Data are normalized with β-actin values and are expressed as fold changes of β-actin. All experiments were repeated at least three times. Data are means ± SEM. (n = 3) (Dunnett’s test, *P<0.05 *vs.* control).

The ERK pathway is frequently up-regulated in a variety of cancers [29], [30], and there is some evidence showed that ANXA1 modulates the ERK pathway at a proximal site [31]. To further investigate a potential mechanism that could be involved in the growth inhibition and pro-apoptosis in cancer cells, the expression of ERK and phosphorylated ERK (pERK) was detected. pERK was significantly decreased in siANXA1-transfected cells compared with siMock group. The results demonstrated that the ERK pathway was attenuated in siANXA1-transfected cells (Fig. 5).

## Discussion

Prostate cancer is the second most commonly diagnosed cancer and the second leading cause of cancer deaths among men. Currently, several effective radiation and surgical therapies can be offered for the clinical treatment of prostate cancer; however, therapies for prostate cancer are far from satisfactory and advanced prostate cancer remains incurable [9]. There is an urgent need to find substances or drugs that are suitable for the prevention and better control of prostate cancer. NaB, a member of the group of histone deacetylase inhibitors (HDACs), is a naturally occurring short-chain fatty acid that is capable of initiating differentiation of many cell types, such as HT29 human colon cancer cells and gastric cancer cells [32], [33]. NaB could increase antitumor activity with less toxicity, and there are several lines of evidence that have demonstrated that NaB can induce growth inhibition and apoptosis in numerous cancers, including prostate cancer [5], [6], [9]. However, the exact mechanism of NaB in tumorigenesis is poorly understood.

It is now well established that NaB can up-regulate the expression of ANXA1. Moreover, annexins are frequently dysregulated in various cancer types and their expression has indicated a tumor suppressor or possible homeostatic role [34], [35]. Thus, we assumed that NaB inhibited the cell proliferation and induced apoptosis through the up-regulated expression of ANXA1.

In the current study, we evaluated the proliferation inhibition and apoptosis promotion effects of NaB in human prostate cancer DU145 cells, along with its mechanism of action. Our studies showed that NaB at 1–10 mmol could both inhibit DU145 cell proliferation and induce cell apoptosis. Apoptosis, which is also known as programmed cell death, is a physiological process which is regulated by a number of well-characterized genes and is important for the development and homeostasis of many organisms. In this study, we found that NaB can down-regulate bcl-2 expression and up-regulate Bax expression. It is likely that NaB induces prostate cancer DU145 cell apoptosis by inhibiting bcl-2 expression and activating Bax.

Simultaneously, we observed that NaB up-regulated the expression of ANXA1 in DU145 cells. To determine whether ANXA1 function is relevant to NaB action, we performed functional studies using siRNA. The results showed that inhibition of the expression of ANXA1 significantly decreased the NaB action of inhibiting cell growth and pro-apoptosis. Moreover, our results suggested that the mechanism of NaB against apoptosis through the up-regulation of ANXA1 was related to the ERK signal pathway. Inhibiting the expression of ANXA1 could inhibit the phosphorylation of ERK1/2, which indicates that the expression of ANXA1 could activate the ERK signal pathway. To date, the expression of ANXA1 being up-regulated by NaB in prostate cancer has not been confirmed. In our study, we provided some evidence that the NaB up-regulates the expression of ANXA1 and is correlated with the progression of prostate cancer. These facts strongly imply that ANXA1 has an important role in prostate cancer progression.

In conclusion, NaB exhibits anti-proliferative and pro-apoptotic activity in a dose-dependent manner in human prostate cancer cells. Furthermore, NaB can increase the expression of ANXA1, and ANXA1 can increase the activity of ERK signaling pathway. Further studies are needed to reveal the definite interactions of ANXA1 and the ERK signaling pathway. The present findings suggest that NaB is particularly effective in inhibiting the progression of prostate cancer via the up-regulation of the expression of ANXA1.
